# The Effects of *Nigella Sativa* Hydro-alcoholic Extract on Memory and Brain Tissues Oxidative Damage after Repeated Seizures in Rats

**Published:** 2015

**Authors:** Farzaneh Vafaee, Mahmoud Hosseini, Zahra Hassanzadeh, Mohammad Amin Edalatmanesh, Hamid Reza Sadeghnia, Masoumeh Seghatoleslam, Seyed Mojtaba Mousavi, Atefeh Amani, Mohammad Naser Shafei

**Affiliations:** a *Neurogenic Inflammation Research Center, Department of Physiology, School of Medicine, Mashhad, University of Medical Sciences, Mashhad, Iran. *; b *Neurocognitive Research Center, Department of Physiology, School of Medicine, Mashhad, University of Medical Sciences, Mashhad, Iran. *; c *Department of Physiology, College of Science, Shiraz Branch, Islamic Azad University, Shiraz, Iran. *; d *Pharmacological Research Center of Medicinal Plants, School of Medicine, Mashhad University of Medical Sciences, Mashhad, Iran. *; e *Department of Anatomy and Cell Biology, School of Medicine, Mashhad University of Medical Sciences, Mashhad, Iran.*

**Keywords:** *Nigella sativa*, Pentylenetetrazole, Repeated seizures, Oxidative stress, Memory

## Abstract

Regarding the therapeutic properties of *Nigella sativa* (NS), the effects of the plant hydro – alcoholic extract on learning, memory and brain tissues oxidative damage were investigated in penthylenetetrazole (PTZ) - induced repeated seizures.

There were 4 experimental groups including: 1- control group; received saline, 2- PTZ group ; received saline and PTZ (50 mg/Kg, *i.p*) , 3-PTZ- NS 200 and 4- PTZ- NS 400 ; received 200 and 400 mg/Kg of NS extract respectively, before PTZ injection in 5 consecutive days. Seizure scores were lower in PTZ – NS 200 and 400, furthermore the seizure onset latencies were higher in these groups than PTZ group (P<0.05 and P<0.01 ). In Morris water maze, the time spent in target quadrant by PTZ group was lower than control group (P<0.05); while, 400 mg/Kg of the extract increased it (P<0.01). In the passive avoidance test, delay time to enter the dark by PTZ group was lower than control at 1 and 24 hours after training (P<0.01- P<0.001); while, 400 mg/Kg of the extract increased it (P<0.05). The total thiol concentration in hippocampal and cortical tissues of PTZ group was reduced while, MDA concentration was higher than control (p<0.05 - p<0.001). Administration of the extract increased the total thiol and decreased the MDA concentrations (p<0.01- p<0.001).

It is concluded that the hydro-alcoholic extract of NS possess beneficial effects on learning and memory impairments in repeated seizures model which is accompanied by antioxidant effects in the brain.

## Introduction

Neuronal hyperexcitability and excessive production of free radicals have been implicated in the pathogenesis of a considerable range of neurological disorders, including epilepsy. The increased susceptibility of the brain to oxidative damage highlights the importance of understanding the role of oxidative stress in the pathophysiology of seizures and epilepsy (1). 

Furthermore, accumulating evidence revealed the importance of oxidative stress as a consequence epileptic seizures (1). It has been found that the prolonged seizures are followed by oxidative damage to lipids, DNA, and susceptible proteins (2). It has also been well documented that oxidative damage plays an important role in the pathogenesis and complications of other central nervous system (CNS) disorders such as learning and memory impairments while, antioxidants have been shown to prevent memory impairments in various experimental models (3, 4). 

Patients with epilepsy frequently manifest cognitive and affective disorders such as spatial memory deficit and impaired emotional learning (5) but according to other studies, the spatial memory has remained intact after recurrent seizures (6). Most obviously, the experiments have already confirmed that seizures are accompanied with oxidative damage to the parts of the brain such as the hippocampus and other parts of the limbic system (7). However, experimental evidence indicates that antioxidant compounds can protect against the neuronal damage observed during epilepsy. Studies have demonstrated that ascorbic acid can ameliorate oxidative stress in the hippocampus during seizures (7). It has been reported that even a brief seizure induced by a single injection of PTZ also impairs learning and memory (8).

*Nigella sativa L.* (NS) is an annual flowering plant native to different regions of southern Europe and some parts of Asia. The flowers are delicate and are usually colored pale blue and white with small black seeds (9). In traditional medicine, this herb is known for having a healing power so that it has been used in the Middle East and Far East for treating diseases such as asthma, headache, dysentery, infections, obesity, back pain, hypertension and gastrointestinal problems (9, 10). The results of previous experimental studies have supported the pharmacological effects of the seeds of NS or thymoquinone (9). It has been reported that the extract of NS seeds and thymoquinone showed the beneficial effects in a depression like behavior in rats which was accompanied with the level of serotonin in the brain (10, 11). The beneficial effects of NS on learning, memory and cognition in human and animals models have been reported (12-15). In traditional medicine, NS has been suggested to be effective against epilepsy (16). The results of human and animal studies have shown that NS and thymoquinone have antiepileptic properties (16-22).

The anti- oxidant effects of *Nigella sativa* and thymoquinone has been suggested (23, 24). Hosseinzadeh *et al.* (25) found that *Nigella sativa* oil and thymoquinone have antioxidant effects during cerebral ischemia-reperfusion injury in rat hippocampus (25). The antioxidant effects of NS in other animals models of nervous system disorders have also been reported which sometimes have been confirmed by human studies (21, 26, 27). 

Regarding the role of brain tissues oxidative damage in memory impairments due to repeated seizure and based on the beneficial effects of NS on both seizures and memory impairments and the antioxidant effects of the plant which have been reported in traditional medicine and in the experimental studies, the present study was designed in order to evaluate possible effects of hydro-alcoholic extract of NS on brain tissues oxidative damage and learning and memory in PTZ induced repeated seizures in rats.

## Experimental

*Preparing the plant extract*

Powdered seeds (100 g) of NS were extracted in a Soxhlet extractor with ethanol (70%). The resulting extract was concentrated under reduced pressure and kept at -20 ^o^C until being used (yielded 32%). The extract was dissolved in saline (4, 28).

*Animals and the experimental protocol*

Forty male Wistar rats (8 weeks old, 230 ± 20 g) were kept at 22 ± 2 ^o^C and 12 h light/dark cycle, started at 7:00 am. They were randomly divided to four groups and treated according to the experimental protocol. All measurements were performed between 10 and 14 am. 

Group 1 (control group) received saline instead of NS extract or PTZ. The animals in group 2 (PTZ group) were treated by saline and injected by PTZ (50 mg/Kg, *ip*). Groups 3-4 (PTZ-NS 200) and (PTZ-NS 400) were treated by 200 and 400 mg/Kg of NS (*ip*) before each PTZ injection respectively. 

*PTZ-induced repeated seizures*

The animals were injected by 50 mg/Kg PTZ. Following each injection the rats were placed in a Plexiglas cage separately, and observed for 60 minutes. The resultant seizures were classified according to a modified Racine scale as follows: 1 － Mouth and facial movements; 2 － Head nodding; 3 － Forelimb clonus; 4 － Rearing; 5－ Rearing and falling. The latency to the first sign of seizure and duration of seizures were also recorded (28). 

*Morris water maze test*

To evaluate spatial memory, the rats were tested in Morris water maze which is a black circular pool of 150 cm diameter and 60 cm height, filled with 24 – 26 °C water to a depth of 30 cm. It is divided geographically into four quadrants of North, East, South and West. In the center of the southeast quadrant, a circular Plexiglas platform (10 cm in diameter and 28 cm in height) was located, hidden 2 cm below the surface of water. 

Before starting the experiments, each rat was handled daily for 3 days and then the rats were accustomed to the water maze for 30 s without a platform.

Each rat participated in 12 trials, which were organized into three blocks of four trials (1 trial/start position within a block). Each block was considered as a separate test session and the blocks were separated by 30 min intervals. The rats were randomly released in one of the four quadrants of the pool while facing the wall of the tank. On each trial, the rat was allowed to swim until it found and remained on the platform for 20 s. If 60 s had passed and the animal had not found the platform, it was guided to the platform and allowed to stay on the platform for 20 s. During all these stages a camera was installed above the pool to detect the pathway of the rats which was then received by a computer and recorded. The time latency to reach the platform and also the length of swimming path were compared among groups. In the retention phase (24 h later), the platform was removed and a 60-s probe trial was conducted to examine how well the rats had learned the exact location of the platform. The platform was removed, and the animals were allowed to swim for 60 seconds. The time spent in the target quadrant (Q1) was compared among groups (3, 29, 30). 

*Passive avoidance test*

The passive avoidance learning test based on negative reinforcement was carried out. The apparatus had a grid floor and comprised of two compartments: one dark and the other one lighted, with a small gate which connected these two parts. This test is performed with the knowledge that rats have a native preference to the dark environment. Before beginning the training session, the animals were familiarized with the apparatus for two successive days (5 min per day). On the ensuing day, they were placed in the lighted compartment and the time latency for entering the dark compartment, as well as the time spent in dark and light compartments were noted down. During the training phase, the animals were located in the lighted compartment while facing toward the walls and away from the gate and received an electric shock (1.5 mA, 2 s duration) when they were entered the dark part. The animals were then returned to their cages. In the retention or test phase, carried out at one and twenty four hours after the training sessions, the rats were placed in the light compartment and time latency to enter the dark compartment as well as the time spent by the animals in the dark and light compartments were recorded (31). 

*Biochemical assessment*

The animals were sacrificed after behavioral study, the brains were removed and dissected on an ice-cold surface and conserved for biochemical measurements.

Total thiol groups were measured using DTNB as a reagent. This reagent reacts with the thiol groups to produce a yellow colored complex which has a peak absorbance at 412 nm (Ellman, 1959). Briefly, 1 mL Tris-EDTA buffer (pH=8.6) was added to 50 μL brain homogenates in 1ml cuvettes and the sample absorbance was read at 412 nm against Tris-EDTA buffer alone (A1). Then 20 μL DTNB reagent (10 mM in methanol) was added to the mixture and (stored in laboratory temperature) the sample absorbance was read again after 15 minutes (A2). The absorbance of DTNB reagent was also read as a blank (B). Total thiol concentration (mM) was calculated from the following equation (3).

Total thiol concentration (mM) = (A2-A1-B) × 1.07/0.05 × 13.6

Malondialdehyde (MDA) level , as an index of lipid peroxidation, also was measured. MDA reacts with thiobarbituric acid (TBA) as a thiobarbituric acid reactive substance (TBARS) to produce a red colored complex which has peak absorbance at 535 nm. 2 mL from reagent of TBA/TCA/HCL was added to 1 mL of homogenate and the solution was heated in a water bath for 40 min. After cooling, the whole solution was centrifuged by 1000 g for 10 min. The absorbance was measured at 535 nm. The MDA concentration was calculated as follows: C(m) = Absorbance/( 1.65 × 10^5^)(3).

*Statistical analysis*

The data were expressed as mean ± SEM. To compare the time latency, traveled path length and speed in water maze, two way ANOVA was used. To analyze the data of passive avoidance test as well as biochemical measurements, one-way ANOVA followed by a post hoc comparison test was used. The criterion for the statistical significance was set at *p* < 0.05. 

## Results

*PTZ-induced repeated seizures*

**Figure 1:**
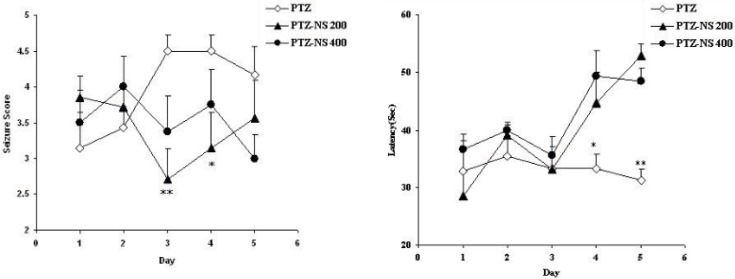
Comparison of seizure score (A) and latency to the onset of seizures (B) between groups. Data are presented as mean ± SEM (n = 9-10 in each group). ^*^p<0.05 and ^**^p<0.01 compared to PTZ group. The animals were injected by PTZ and observed for 60 minutes. The animals of PTZ- NS 200 and PTZ- NS 400 groups were treated by 200 and 400 mg/Kg of *Nigella sativa *(NS) extract before PTZ injection. The animals of PTZ group received saline instead of NS extract.

The seizure scores in both PTZ - NS 200 and PTZ- NS 400 groups were significantly lower than PTZ group on days 3 and 4 (P<0.01 and P<0.05 respectively; Figure 1 A). The latency to the onset of seizures was also significantly higher than PTZ group on days 4 and 5 (P<0.05 and; P<0.01 Figure 1 B). 

*Morris water maze test*

**Figure 2:**
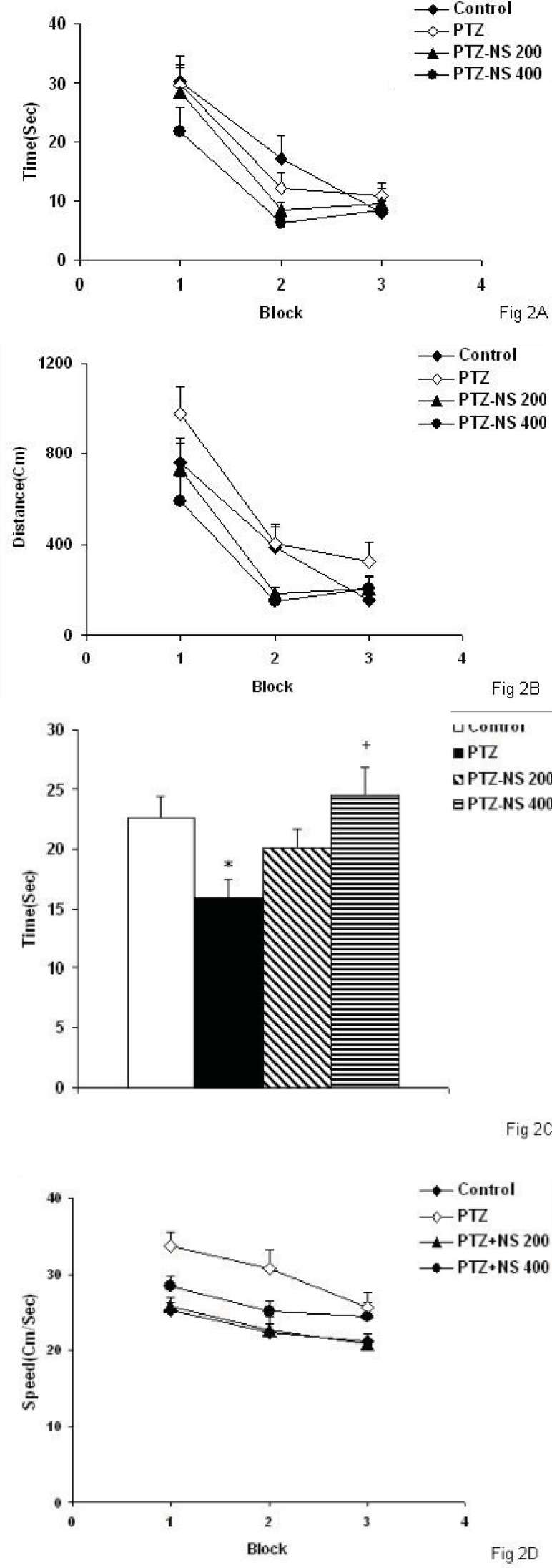
Comparison of time latency (A), the length of swimming path (B) to reach the platform and the time spent (C) in target quadrant (Q1) between groups. The comparison of speed was also shown in (D). Data are presented as mean ± SEM (n = 9-10 in each group).

There was no significant differences between PTZ and control groups neither in escape latency nor in traveled path (Figures 2A and 2B). There was no significant difference in time latency to reach the platform when the animals of the PTZ-NS 200 and PTZ – NS 400 groups were compared with PTZ group (Figure 2A). The traveled distance to reach the platform in both PTZ-NS 200 and PTZ – NS 400 groups were not different from PTZ group (Figure 2B; s). In the probe trial, the time spent in the target quadrant (Q_1_) by the animals of PTZ group was significantly lower than control group (Figure 2C, *p*<0.05). The animals of PTZ-NS 400 group spent more time in target quadrant compared with PTZ group (Figure 2C, *p*<0.05) however, there was no significant difference between PTZ-NS 200 and PTZ groups. The speed of the animals of PTZ group was not significantly higher than that of control group (Figure 2D, *p*<0.001).Treatment by both 200 and 400 mg/Kg of the extract reduced the speed to the animals to reach the platform compared to PTZ group but the effects were not significant (Figure 2D). 

There was no significant difference between PTZ and control groups neither in escape latency nor in traveled path. There was no significant difference in time latency to reach the platform when the animals of the PTZ-NS 200 and PTZ – NS 400 groups were compared with PTZ group. The traveled distance to reach the platform in both PTZ-NS 200 and PTZ – NS 400 groups were lower than PTZ group.

^*^ *p*<0.05 compared to Control group, ^+^*p*<0.05 compared to PTZ group. The animals of PTZ- NS 200 and PTZ- NS 400 groups were treated by 200 and 400 mg/Kg of NS extract before PTZ injection. The animals of PTZ group received saline instead of NS extract. 

*Passive avoidance test*

**Figure 3:**
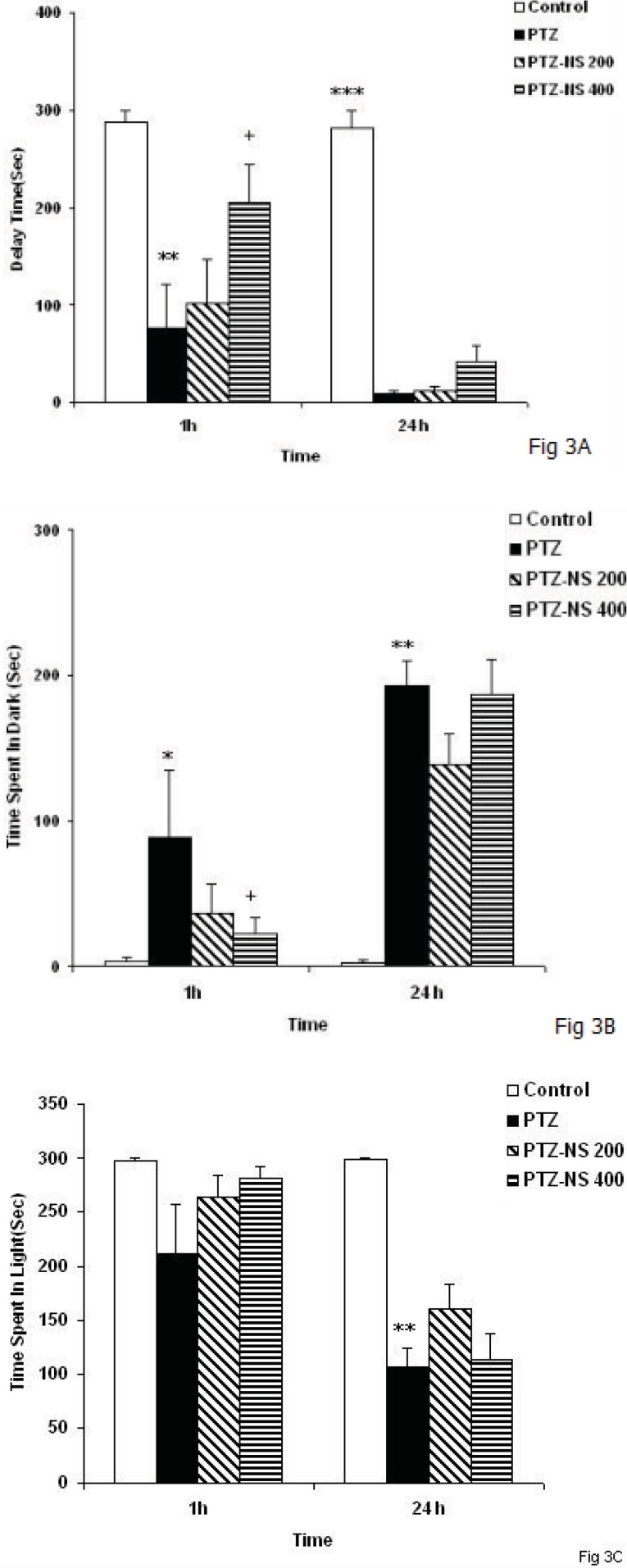
Comparison of time latency for entering the dark compartment(A), the total time spent in the dark compartment(B), the total time spent in the light compartment (C) at 1 and 24 h after receiving the shock in the experimental groups. Data are presented as mean ± SEM (n = 9-10 in each group). ^*^p<0.05, ^**^p<0.01 and ^***^p<0.001 compared to control group, ^+^*p*<0.05 compared to PTZ group. The animals were injected by PTZ and observed for 60 minutes. The animals of PTZ- NS 200 and PTZ- NS 400 groups were treated by 200 and 400 mg/Kg of *Nigella sativa* extract before PTZ injection. The animals of PTZ group received saline instead of *Nigella sativa* extract.

**Figure 4:**
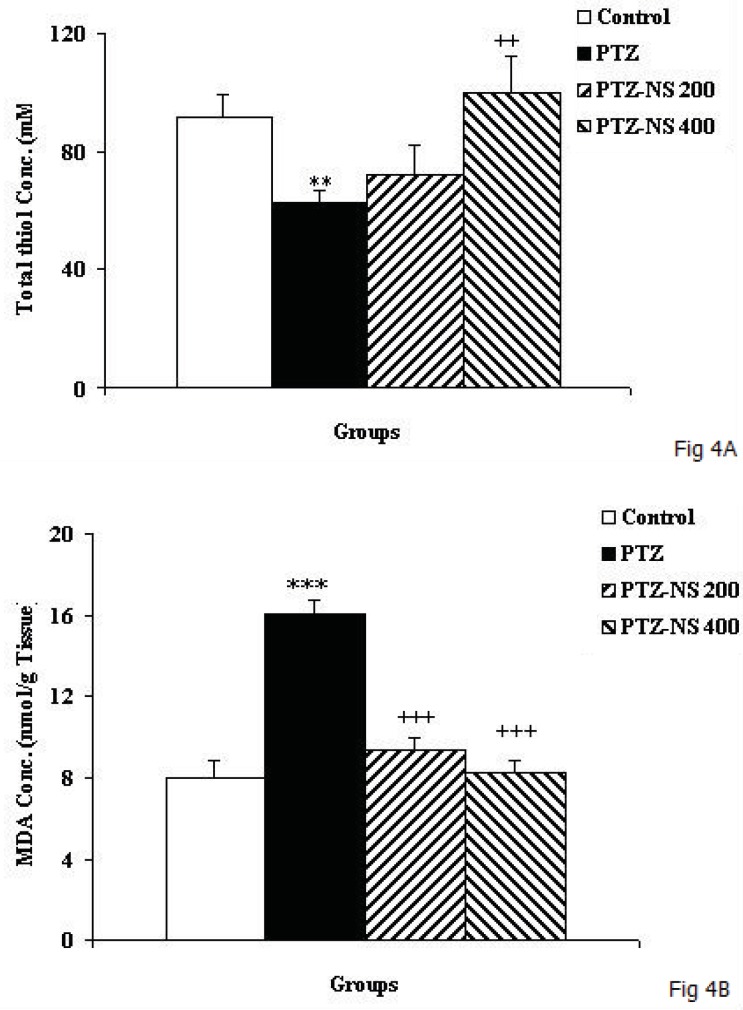
The total thiol concentration (A) and MDA concentration (B) in hippocampal tissues of 4 groups. Data are shown as mean ± SEM of 9-10 animals per group.

^**^ *p*<0.01 and ^***^p<0.001 compared to control group, ^++^*p*<0.01 and ^+++^*p*<0.001 compared to PTZ group.

The time latency for entering the dark compartment was lower in the PTZ group than control group 1 h after taking a shock (Figure 3A, *P < *0*.*01). Treatment of the animals by 400 mg/Kg of NS extract significantly increased the time latency for entering the dark compartment 1 h after receiving a shock (Figure 3A, *P < *0*.*05). However, there was no significant difference between PTZ-NS 200 and PTZ groups (Figure 3A). The time latency for entering the dark compartment in PTZ treated rats was lower than control group 24 h after the shock (Figure 4A, *P < *0*.*001). Administration of 400 mg/Kg of the NS extract increased non-significantly the time latency for entering the dark compartment 24 hours after shock (Figure 3A). The time latency for entering the dark compartment didn’t change by 200 mg/Kg of the extract 24 h after taking a shock (Figure 3A).

One and twenty-four hours after receiving the shock, the total time spent in the dark compartment by the animals of the PTZ group was more than control group(Figure 3B, *P < *0*.*05 and *P < *0*.*01 respectively). There was no significant difference between PTZ and PTZ- NS 200 groups in the total time spent in the dark at 1 or 24 hours after the shock (Figure 3B). Pretreatment by 400 mg/Kg of the extract reduced the total time spent in the dark at both 1 and 24 hours after receiving a shock (Figure 3B, *P < *0*.*05) however, 400 mg/Kg of the extract didn’t change the total time spent in the dark.

The results also showed that the time spent in the light compartment by the animals of PTZ group was lower than control group 24 h after receiving a shock (Figure 3C,* P < P < *0*.*01) however, there was no significant difference at 1 h after shock. Treatment of the animals by NS extract didn’t affect the time spent in the light compartment when the animals were examined at 1 and 24 h after receiving a shock (Figure 3C). 

*Biochemical assessment results*

The total thiol concentration in hippocampal tissues of PTZ group was significantly lower than control animals (Figure 4A*; p*<0.01). In PTZ –NS 400 group, the hippocampal total thiol concentration was significantly higher than PTZ group (Figure 4A;* p*<0.01) however, 200 mg/Kg of the extract didn’t show any effect. MDA concentration in hippocampal tissues of PTZ treated animals was higher than control ones (Figure 4B; *p*<0.001). Pretreatment of the animals by both 200 and 400 mg/Kg of NS extract decreased the MDA concentration in hippocampal tissues compared to PTZ group (Figure 4B; both *p*<0.001). 

**Figure 5:**
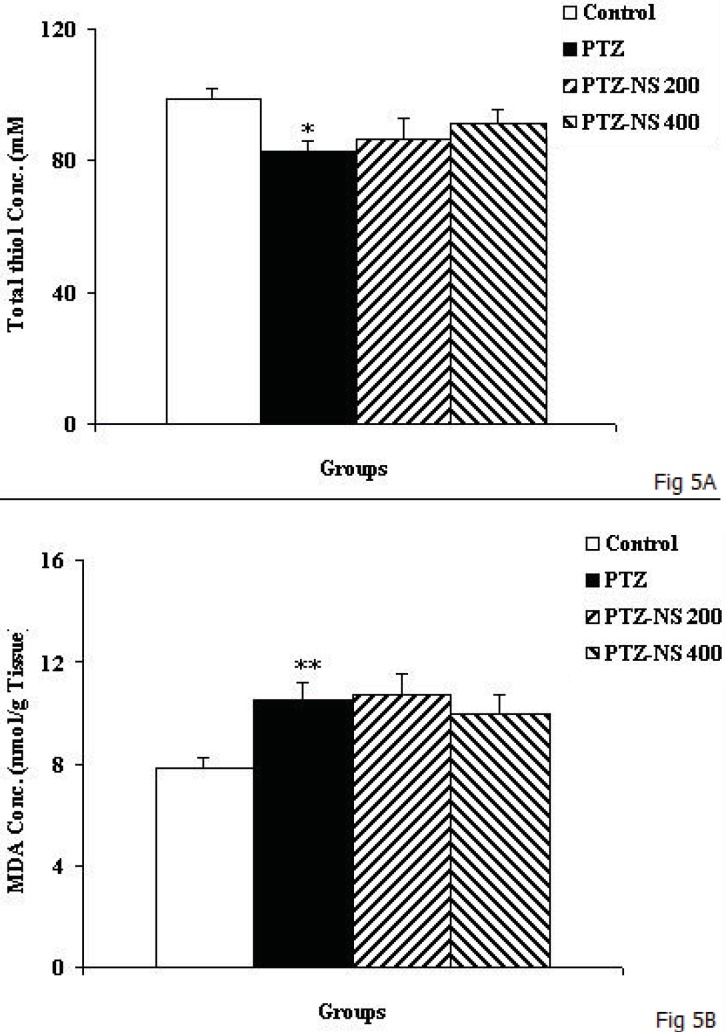
The total thiol concentration (A) and MDA concentration (B) in cortical tissues of 4 groups. Data are shown as mean ± SEM of 9-10 animals per group. ^*^p<0.05 and ^**^p<0.01 compared to Control group.

As the Figure 5A shows, the total thiol concentration in cortical tissues of PTZ group was significantly lower than control animals (Figure 5A; *p*<0.05). Administration of NS extract before each PTZ injection didn’t affect the total thiol concentration (Figure 5A). The results also showed that cortical MDA concentration in PTZ group was significantly higher than control group (Figure 5B*; p*<0.01). There was no significant difference in MDA concentration of cortical tissues among PTZ-NS 200, PTZ-NS 400 and PTZ groups (Figure 5B).

## Discussion

The results of the present study showed that PTZ- induced repeated seizures impaired learning and memory and also induced brain tissue oxidative damage in rats which was reduced by the NS extract. Oxidative stress is a basis for many neurological and neurodegenerative disorders. Oxidative stress is proposed to have a role in the pathogenesis of epilepsy (1). The presence of a high level of reactive oxygen species (ROS), including superoxide anions, hydroxyl radicals and hydrogen peroxide in the brain after seizures may confirm this idea(1). However, it has been also well documented that brain tissue oxidative damage has a role in the pathogenesis of the consequences of epilepsy (2). Accordingly, in the present study an increase in MDA level was observed which was accompanied by a reduction in total thiol groups in the brain tissues of animals subjected to PTZ-induced seizure. The elevation of ROS in the brain may lead to brain tissue oxidative damage which consequently causes problems such as depression, anxiety and memory loss (32). The results of the present study confirmed that elevation of MDA and decreasing of thiol group concentrations in brain tissues of PTZ treated rats were accompanied with learning and memory impairments. Some study results supported learning and memory decline in epileptic persons (5). Using kindled animals, it has been repeatedly reported that prolonged and recurrent seizure impairs learning and memory (33). The results of the present study showed that repeated seizures induced by injection of PTZ for 5 consecutive days also lead to memory impairments. It is also suggested that the effect of repeated seizure isn’t due to its effect on motor activity because its effect on swimming speed was positive in rats but it had a negative effects on memory criteria when the animals were examined in prob test to remember the location of the platform. Regarding the results of the present study the brain tissue oxidative stress as a link between seizures and memory impairments might be suggested. 

The seed extract of NS has been used as a natural remedy for treating of a number of illnesses and conditions for many years (9). The effects of NS on learning and memory have also been suggested. NS oil enhanced learning and memory abilities of the rats when examined using eight-arm radial arm maze (14). Using Morris water maze also, NS improved learning and memory impairments due to global cerebrovascular hypoperfusion in rats (12). Protective effects of Nigella sativa against diabetes induced learning and memory impairment has also been reported (15). Results from human studies also confirmed the beneficial effects of NS on memory, attention and cognition without any side effects on kidney, heart and liver (13) 

Mohamed *et al*. (2005) showed that treatment of rats with thymoquinone prevented the experimental autoimmune encephalomyelitis (34), which was probably due to its antioxidant and anti-inflammatory properties (35). The antioxidant effects of NS and thymoquinone have been repeatedly reported to be comparable to the effects of vitamine E and vitamine C (23, 25). However it was not compared to other well-known antioxidants, the results of the present study also showed that pretreatment by NS extract prevented elevation of MDA brain tissue concentration while it enhanced total thiol concentration. 

Moreover, it has been previously reported that hippocampal neurodegeneration after chronic toluene exposure in rats was prevented by* NS* oil and thymoquinone (36). Hosseinzadeh *et al.* have also shown that NS and thymoquinone prevented lipid peroxidation increment in hippocampal proteins following global cerebral ischemia-reperfusion injury model in rats (25). The anti-Parkinson’s activity of NS was also accompanied with the antioxidant effects (27, 37). The antioxidant effects of NS oil was comparable to the effects of valproate in PTZ induced kindled mice (26). Antiepileptic effect of NS and its ability to inhibit excessive ROS formation in PTZ - induced seizures has been reported (26). A neuroprotective effect of NS in an experimental model of spinal cord injury in rats has also been attributed to its antioxidant capability (38). It has been shown that NS extract and thymoquinone attenuated oxidative stress and neuropathy in diabetic rats (39). Regarding these reports and the results of present study, it seems that NS attenuates both seizures severity and brain tissues oxidative damage and protects learning and memory impairments in rats. It does not seem that the effect of the extract be due to its effect on motor activity because both doses of the extract reduced the speed compared to PTZ group but they enhanced the time spent in target quadrant in prob test.

NS was also found that modulates the brain content of excitatory and inhibitory neurotransmitters such as aspartate and glutamate, GABA and glycine (40). NS increased GABA released from cultured neurons whereas secretion of glutamate, aspartate, and glycine were decreased. (41). Regarding these findings and the sedative and depressive effects of NS observed *in-vivo*, the changes of inhibitory/excitatory amino acids levels may have a role in the effects which was seen in the present study. The effects of NS and thymoquinone on pentobarbital-induced hypnosis, locomotor activity and motor coordination which have been previously reported might also be conceivable (18). 

Hosseinzadeh and Parvardeh using a PTZ model showed that thymoquinone prolonged the onset of seizures and reduced the duration of myoclonic seizures and reduced mortality rate however, in maximal electroshock (MES) model, thymoquinone failed to reduce the duration of seizure, whereas exhibited a complete protection against mortality. In this study the interactions with opioid and GABAergic systems were suggested as possible mechanisms (18). However, thymoquinone didn’t show the protective activity on percentage of protection of animals against MES- induced tonic seizures in another study (42). Thymoquinone has been reported to have anti-epileptic effects in children with refractory seizures (19). In another study NS aqueous extract, and its volatile oil and hymoquinone, showed antiepileptic effects using PTZ induced seizures model and potentiated the effects of valproate (43). Nigella sativa oil reduced the sensitivity of kindled mice to the convulsive effects of PTZ and mortality rate while, valproate was ineffective in preventing development of any of these effects (26). It was also shown that thymoquinone in both PTZ and MES- induced seizure models increased sodium valproate potency and also reduced the hepatotoxic effects of sodium valproate (44)**.**

Furthermore, it has been postulated that *Nigella sati*v*a *oil and thymoquinone produced antinociception in the formalin test by releasing endogenous opioid peptides in the CNS, and causes an antinociceptive tolerance following repeated administration (45). It was also shown that the antinociceptive effect of morphine was reduced in thymoquinone and NS oil-pretreated mice (45). The ability of the hydroalcoholic extract of NS to change morphine induced conditioning place preference may also confirm the interaction of the plant with opioid system (46). Therefore, it might be suggested that NS and its ingredients have interaction with opioid system to reduce seizure severity and prevent memory impairments which was seen in the present study but it needs to be more investigated. 

As it was mentioned NS is containing many compounds, the main of them is thymoquinone. In the present study the ingredient(s) responsible for the effects of the extract was not known and it needs to be investigated in future. Recently, it is reported that besides thymoquinone, the main ingredient which has an important role in pharmacological effects of NS, other component(s) may also be involved in the results of the present study. More recently, melanin has been shown to occur abundantly in the seed coats of NS (47). On the other hand, according to a number of botanical sources, melanin has been found to act as an immunomodulator (48, 49). Therefore, involvement of this ingredient of NS may also be suggested as a possible compound which plays a role in the result of the present study. 

## Conclusion

The results of the present study showed that the hydro-alcoholic extract of *Nigella sativa* prevented learning and memory impairments as well as brain tissue oxidative damage after PTZ - induced repeated seizure in rats. These results support the traditional belief about the beneficial effects of *Nigella sativa* on the nervous system. Further studies are required for determining (confirming) the protective effect of *Nigella sativa*.
